# SFD-ADNet: Spatial–Frequency Dual-Domain Adaptive Deformation for Point Cloud Data Augmentation

**DOI:** 10.3390/jimaging12020058

**Published:** 2026-01-26

**Authors:** Jiacheng Bao, Lingjun Kong, Wenju Wang

**Affiliations:** 1 College of Publishing, University of Shanghai for Science and Technology, Shanghai 200093, China; 233352870@st.usst.edu.cn; 2 Printing and Packaging Engineering Department, Shanghai Publishing and Printing College, Shanghai 200093, China; klj@sppc.edu.cn

**Keywords:** point cloud robustness, data augmentation, sequential spatial features, adaptive bidirectional Mamba, frequency-domain multi-scale features, multi-domain feature fusion

## Abstract

Existing 3D point cloud enhancement methods typically rely on artificially designed geometric transformations or local blending strategies, which are prone to introducing illogical deformations, struggle to preserve global structure, and exhibit insufficient adaptability to diverse degradation patterns. To address these limitations, this paper proposes SFD-ADNet—an adaptive deformation framework based on a dual spatial–frequency domain. It achieves 3D point cloud augmentation by explicitly learning deformation parameters rather than applying predefined perturbations. By jointly modeling spatial structural dependencies and spectral features, SFD-ADNet generates augmented samples that are both structurally aware and task-relevant. In the spatial domain, a hierarchical sequence encoder coupled with a bidirectional Mamba-based deformation predictor captures long-range geometric dependencies and local structural variations, enabling adaptive position-aware deformation control. In the frequency domain, a multi-scale dual-channel mechanism based on adaptive Chebyshev polynomials separates low-frequency structural components from high-frequency details, allowing the model to suppress noise-sensitive distortions while preserving the global geometric skeleton. The two deformation predictions dynamically fuse to balance structural fidelity and sample diversity. Extensive experiments conducted on ModelNet40-C and ScanObjectNN-C involved synthetic CAD models and real-world scanned point clouds under diverse perturbation conditions. SFD-ADNet, as a universal augmentation module, reduces the mCE metrics of PointNet++ and different backbone networks by over 20%. Experiments demonstrate that SFD-ADNet achieves state-of-the-art robustness while preserving critical geometric structures. Furthermore, models enhanced by SFD-ADNet demonstrate consistently improved robustness against diverse point cloud attacks, validating the efficacy of adaptive space-frequency deformation in robust point cloud learning.

## 1. Introduction

Three-dimensional point cloud data augmentation refers to the systematic expansion of the original point cloud through geometric transformations, noise perturbations, or generative modeling while preserving the core semantic information of the point cloud. Its primary objective is to alleviate the problem of unbalanced data distribution and sample scarcity, thereby assisting the model in learning a more robust and generalized 3D feature representation. This technology has been widely implemented in key fields such as autonomous driving [1], robot perception, 3D reconstruction, and cultural heritage [2], providing strong support for intelligent systems to perceive the 3D world. However, although existing data augmentation methods—such as FIXED [3], PolarMix [4], RandomFusion [5], Real3D-Aug [6], PatchAugment [7], IPC-Net [8], Test-Time Augmentation [9], and 3D-VDNet [10]—have demonstrated preliminary effectiveness, they still suffer from two common limitations. First, most of these methods do not take the structure of the downstream classifier into account and often apply geometrically inconsistent transformations, which may produce augmented samples that do not meaningfully improve model robustness. Second, these approaches generally lack the ability to decouple and explicitly control local geometric details and global topological structures, making it difficult to simultaneously learn stable global representations and robust local features. These observations indicate that existing augmentation strategies typically lack an adaptive mechanism capable of jointly decoupling and controlling local details and global topology to achieve both structural fidelity and controllable sample diversity. In particular, under diverse and complex real-world conditions, designing an augmentation framework that is both efficient and robust—while effectively balancing sensitivity to local geometry and consistency of global structure—remains an open and important research problem.

To address these challenges, this paper proposes SFD-ADNet, an adaptive 3D point cloud data augmentation framework that jointly models the spatial and frequency domains through learnable deformation prediction. By integrating serialized spatial features with adaptive multi-scale frequency-domain representations, SFD-ADNet enables coordinated sampling of local details and global topology and supports controllable geometric deformation. This design provides an effective solution for enhancing data diversity while preserving geometric consistency. The main contributions of this work are summarized as follows:

1. SFD-ADNet (Spatial–Frequency Dual-Domain Adaptive Deformation for Point Cloud Enhancement) is a point cloud data augmentation method based on spatial–frequency dual domain adaptive deformation prediction. SFD-ADNet integrates spatial sequence features with adaptive multi-scale frequency-domain features to achieve point cloud enhancement through joint spatial–frequency domain modeling. Experimental results demonstrate that SFD-ADNet generates high-quality, diverse augmented samples and significantly enhances the robustness of downstream models on mainstream benchmarks such as ModelNet40-C and ScanObjectNN-C.
2. SFD-ADNet designs a spatial domain adaptive bidirectional Mamba deformation parameter prediction method to enable spatial feature deformation prediction based on long-range dependencies. This method captures global context and local structural variations through a hierarchical sequential point cloud encoder and an adaptive bidirectional Mamba local geometric feature encoder. Combined with a position-aware anchor deformation parameter prediction approach, it generates high-precision, structurally consistent transformation parameters for each anchor. This enables the model to maintain stable feature extraction even under complex conditions such as density variations, noise interference, and geometric jitter.
3. SFD-ADNet proposes a frequency-domain adaptive multi-scale dual-channel deformation parameter prediction method. This method constructs a frequency-domain feature space using self-tuning Chebyshev polynomial bases and obtains global deformation parameters through a multi-scale, dual-channel frequency-domain deformation parameter prediction approach. It preserves point cloud skeleton structures and key topological patterns while significantly reducing high-frequency noise’s impact on geometric consistency, achieving “enhancement as defense”.
4. SFD-ADNet employs an adaptive space-frequency domain deformation fusion modeling approach to dynamically integrate deformation parameters from both domains and generate enhanced samples. Through an adjustable weighting mechanism, it adaptively balances the two deformation results, producing enhanced point clouds with fused multi-domain features. This enables the model to maintain a balance between structural fidelity and sample diversity, thereby generating task-relevant and generalizable point cloud augmentation data.

## 2. Related Work

### 2.1. Representation Learning Foundations for Point Cloud Data Augmentation

With the rapid development of deep neural networks, learning discriminative representations from 3D point clouds has become a central topic in 3D vision research. Early pioneering works such as PointNet [11] and PointNet++ [12] laid the foundation for deep point cloud processing by directly operating on unordered point sets. Building upon these ideas, a series of convolution-based architectures—including PointCNN [13], KPConv [14], FPConv [15], RegGeoNet [16], and Flattening-Net [17]—have been proposed to better encode local geometric structures through learned neighborhood transformations or regularized representations. Meanwhile, other deep models [13,18,19,20,21,22,23] further enhance local context aggregation by designing task-specific hierarchical feature extractors. More recently, attention-based backbones inspired by Transformers [24] have become mainstream in point cloud analysis. Representative approaches such as Point Transformer [25], Fast Point Transformer [26], and Stratified Transformer [27] integrate local interactions with global dependencies and achieve state-of-the-art performance on various 3D understanding benchmarks. Despite their strong effectiveness on specific tasks, these architectures often exhibit limited transferability across heterogeneous tasks and sensing modalities. To alleviate reliance on labeled data and improve generalization, self-supervised pretraining has gained increasing attention. Masked modeling strategies—including Point-BERT [28], Point-MAE [29], MaskPoint [30], and occlusion-based completion methods [31]—enable networks to learn transferable representations from large-scale unlabeled point clouds. Beyond single-modality learning, several approaches leverage cross-modal supervision: CrossPoint [32] and PointVST [33] align point clouds with images, while ACT [34], ULIP-2 [35], and ReCon [36] incorporate language and visual cues to enhance robustness and semantic consistency. However, most of these pretraining frameworks rely on Transformer-based backbones, whose quadratic computational complexity limits scalability when processing long point sequences. This often necessitates coarse block partitioning, potentially leading to the loss of fine-grained geometric details. In contrast, Mamba adopts a state-space modeling paradigm with linear complexity, enabling efficient modeling of long-range dependencies while preserving local geometric fidelity. As a result, it offers a more favorable balance between accuracy and efficiency compared to Transformer-based counterparts.

### 2.2. Spatial-Domain Point Cloud Augmentation Methods

Spatial-domain point cloud augmentation methods aim to enrich training data by directly manipulating point coordinates and geometric attributes, thereby reconstructing spatial distributions and introducing geometric diversity in a structured manner. Early mixing-based strategies primarily combine multiple point clouds using predefined rules such as region exchange, rotation, and point-level fusion. Representative methods include class-balanced PolarMix [37] and keypoint-aware fusion approaches such as FPSMix [38], which improve data diversity and class balance by selectively exchanging or reweighting informative substructures. Although effective in expanding sample distributions and enhancing robustness, these methods often rely on heuristic mixing strategies and lack explicit modeling of intrinsic geometric structures, which may result in physically inconsistent augmented samples. To better preserve geometric plausibility, local and structure-aware augmentation methods guide fine-grained transformations using local neighborhoods or contextual information while attempting to maintain global structural consistency. Some approaches introduce local noise injection, smooth deformation, or context-sensitive geometric transformations to improve robustness against noise, density variations, and partial occlusions [39,40,41]. Others further incorporate surface-aware representations or alignment strategies to stabilize feature extraction under geometric transformations [42,43]. Despite improvements in structural awareness, these methods often struggle to decouple local detail enhancement from global topology preservation. Excessive context aggregation or fixed alignment procedures may limit generalization across diverse scenarios. More recently, to overcome the limitations of manually designed transformations, automated and learning-based augmentation strategies have been proposed. These methods employ learnable deformation predictors, adaptive masks, or curriculum-based augmentation strategies to dynamically generate task-relevant perturbations [44,45,46]. By aligning augmented samples with realistic corruption patterns, they improve adaptability across tasks and data distributions. However, many learning-based strategies still suffer from limited controllability, increased training overhead, or restricted applicability, especially in complex dynamic environments. Consequently, although these methods enhance robustness to data perturbations to some extent, their performance on real-world datasets may remain suboptimal. The adaptive augmentation framework proposed in this paper, SFD-ADNet, is designed to address these limitations.

### 2.3. Spectral-Domain Point Cloud Augmentation Methods

Spectral-domain point cloud augmentation methods transform point cloud data from the spatial domain into the frequency domain using frequency-based representations such as graph Fourier transforms or wavelet transforms. By decomposing geometric information into low-frequency components encoding global structure and high-frequency components capturing local details, these methods enable more fine-grained and controllable manipulation of point cloud features. Early spectral approaches primarily focused on frequency decomposition and constraint-based augmentation. Methods based on graph Fourier analysis or wavelet decomposition introduced frequency-aware regularization or low-frequency constraints to enhance robustness against noise and perturbations, particularly in few-shot or corrupted scenarios [47]. While effective in stabilizing feature representations, these approaches typically rely on limited spectral dimensions and fixed bases, restricting their ability to fully exploit multi-dimensional frequency information or adapt to irregular point cloud distributions. Subsequent studies explored richer spectral modeling and spatio-temporal fusion strategies. By incorporating multi-channel spectral representations and explicitly modeling correlations across frequency bands, these methods improved robustness and representation capacity under various structural variations [48,49]. However, effectively integrating spectral features with spatial information remains challenging, and insufficient fusion between modalities may limit the expressiveness of learned representations. More recent approaches combine spectral processing with attention mechanisms or Transformer-based architectures to jointly capture global context and local geometric details. Multi-scale graph wavelet models with self-attention align learned frequency responses with structural patterns, improving generalization across different scenarios [50]. Other methods employ frequency-domain mixing or balancing strategies to generate samples with diverse frequency distributions, enhancing robustness to multi-scale geometric variations [51,52]. Despite these advances, many spectral-domain methods introduce substantial computational overhead, rely on static spectral bases, or primarily focus on feature robustness rather than explicitly enriching the diversity of original point cloud samples. Overall, compared with spatial-domain augmentation, spectral-domain approaches offer more fine-grained and stable augmentation by explicitly manipulating low- and high-frequency components, thereby improving robustness and generalization. However, existing spectral methods often depend on fixed bases or expensive eigen decomposition, which limits their applicability in dynamic and irregular point cloud augmentation scenarios. These observations motivate the development of an adaptive augmentation framework that jointly leverages spatial structure and learnable Chebyshev polynomial-based spectral representations to achieve efficient, structure-aware, and generalizable point cloud augmentation.

## 3. Materials and Methods

**Figure 1:**
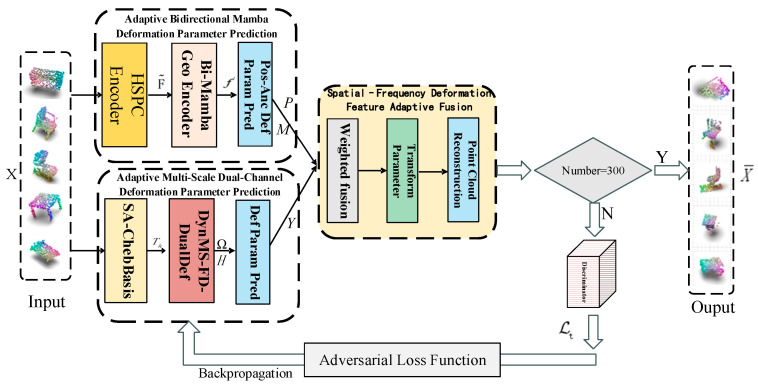
The framework of SFD-ADNet.

The original 3D point cloud sample set is denoted as $X=\{x_{i}\in R^{3}|i=1,2,\ldots ,N\}$, where $x_{i}$ represents the i-th 3D point cloud and N is the total number of point clouds. This point cloud set is used as the input to the proposed model in this paper to generate an augmented point cloud sample set $\bar{X}=\{{\bar{x}}_{i}\in R^{3}|i=1,2,\ldots ,N\}$ that approximates real interference conditions. The SFD-ADNet framework constructed in this paper is shown in Figure 1, which consists of three components: spatial-domain adaptive bidirectional Mamba deformation parameter prediction, frequency-domain adaptive multi-scale dual-channel deformation parameter prediction, and spatial–frequency domain deformation feature adaptive fusion modeling. The spatial-domain adaptive bidirectional Mamba deformation parameter prediction leverages the hierarchical sequential point cloud encoder to extract hierarchical sequential aggregated features $\tilde{F}$ from the original point cloud $x_{i}$. These features $\tilde{F}$ are fed into the adaptive bidirectional Mamba local geometric feature encoder for deep learning of global and local features, outputting geometric aggregated features $f^{\prime }$. Based on $f^{\prime }$, the position-aware anchor deformation parameter prediction method yields mask parameters *M* and spatial deformation parameters *P* (see Section 2.1 for details). The frequency-domain adaptive multi-scale dual-channel deformation parameter prediction constructs a self-tuned Chebyshev polynomial basis from the adaptive graph representation of the original point cloud $x_{i}$. Subsequently, the dynamic multi-scale frequency-domain dual-channel deformation parameter prediction method outputs frequency-domain deformation parameters *Y* (see Section 2.2 for details). In the spatial–frequency domain deformation feature adaptive fusion modeling, the spatial deformation parameters *P*, mask parameters *M*, and frequency-domain deformation parameters *Y* are applied to the point cloud structure through parameter fusion, deformation parameter mapping, and fusion reconstruction. If the model has not reached the preset number of training epochs, the augmented samples, together with the original point clouds, are input into the discriminator [46] to obtain the adversarial loss value $L_{t}$. This loss value further adjusts the generation process of augmented point clouds via the gradient descent backpropagation mechanism (see Section 2.3 for details). Once the model reaches the preset number of training epochs, it can generate the augmented point cloud sample set $\bar{X}$ that realistically simulates real interference conditions.

### 3.1. Spatial-Domain Sequential Feature Encoding and Deformation Control

**Figure 2:**
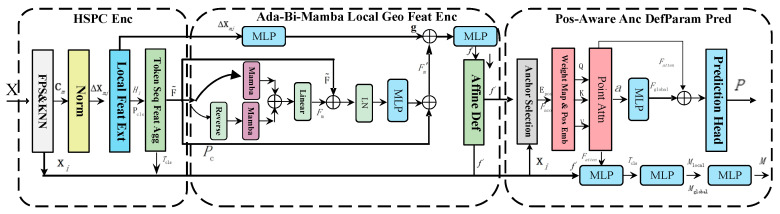
Overview of the spatial-domain sequential feature encoding and deformation control framework.

This module simulates real interference conditions. To achieve high-fidelity and structurally consistent non-rigid deformation of 3D point clouds, the spatial-domain sequential feature encoding and deformation control module is proposed. This module mainly consists of three components: the hierarchical sequential point cloud encoder, the adaptive bidirectional semantic encoder, and anchor-guided deformation parameter prediction, as shown in Figure 2.

#### 3.1.1. Hierarchical Sequential Point Cloud Encoder

To effectively convert unordered 3D point clouds into semantically ordered one-dimensional sequences that can be processed by state-space sequence models such as Mamba, this paper proposes a hierarchical sequential point cloud encoder. The encoder consists of three key steps: ordered point cloud grouping, hierarchical point-wise local feature extraction, and sequential feature aggregation with a learnable CLS token. The overall goal is to transform irregular spatial point sets into a stable and semantically meaningful sequence representation while preserving both local geometry and global structure.

Ordered point cloud grouping: For point cloud data *X*, m central points of neighborhoods $C_{m}$ are selected using farthest point sampling (FPS) [12]. FPS produces an ordered sequence of center points based on progressive farthest-distance selection, which provides a stable and geometry-aware global ordering of the point cloud. Each central point retrieves the nearest k points via the K-nearest neighbors algorithm (KNN) [53] to form local neighborhoods $N_{m}$, as shown in Equation (1).

(1) $$N_{m}=KNN(FPS(X,m),X,k)$$

To achieve translation invariance, the coordinates $x_{i}^{mj}=\{x_{i}^{mj}\in R^{3}|j=1,2,\ldots ,M\}$ of each point within the neighborhoods are normalized with the central point to obtain local relative coordinates $\Delta x_{i}^{mj}=\{\Delta x_{i}^{mj}\in R^{3}|j=1,2,\ldots ,M\}$, as shown in Equation (2).

(2) $$\Delta x_{i}^{mj}=\Delta x_{i}^{mj}-C_{m}$$

Through this process, the original unordered point cloud is mapped into an ordered set of local neighborhoods aligned with the FPS-generated center point sequence, establishing a structured spatial ordering suitable for sequential modeling.

Hierarchical point-wise local feature extraction: The local relative coordinates $\Delta x_{i}^{mj}$ pass through a three-layer point-wise convolution structure, batch normalization (BN) layers, and the SiLU (Sigmoid Linear Unit) activation function to extract point-wise features, which are then aggregated into local features, $H_{l}$ via max pooling, as shown in Equation (3).

(3) $$H_{l}=MaxPool\left(\sigma_{SiLU}\left(BN\left(Conv1D^{3}\left(\Delta x_{i}^{mj}\right)\right)\right)\right)$$

where $\sigma_{Swish}$ denotes the Swish activation function.

Sequential feature aggregation with learnable CLS token: The extracted local features $H_{l}$ are arranged sequentially according to the FPS center point order, forming a one-dimensional feature sequence. To achieve overall feature aggregation while preserving sequence dependencies, a learnable token $T_{cls}$ is randomly initialized and obtains positional embedding parameters $P_{\mathrm{cls}}$ through a positional encoder [54]. The learnable token $T_{cls}$ concatenated in sequence with the local feature sequence $P_{\mathrm{cls}}$ is concatenated in sequence with the local feature sequence $\tilde{F}$ to form the sequential feature, as shown in Equation (4).

(4) $$\tilde{F}=T_{\mathrm{cls}}⊕H_{l}+P_{\mathrm{cls}}$$

where ⊕ denotes the concatenation operation.

#### 3.1.2. Bidirectional Semantic-Aware and Geometry-Adaptive Encoder

Bidirectional Mamba is adopted instead of self-attention to model spectral feature dependencies due to its linear computational complexity and superior efficiency on long sequences. Unlike attention mechanisms whose complexity grows quadratically with sequence length, Mamba-based state-space models enable stable and scalable modeling of long-range dependencies, which is particularly suitable for multi-scale spectral representations with extended-frequency sequences. In addition, the bidirectional design allows for the simultaneous encoding of global structural trends and local high-frequency variations, aligning well with the characteristics of spectral point cloud features. Therefore, a bidirectional semantic-aware and geometry-adaptive encoder is proposed, which consists of three steps: adaptive bidirectional Mamba sequence modeling to generate globally optimized features, residual semantic-enhanced feature generation, and dynamic local geometric feature aggregation.

Adaptive bidirectional Mamba sequence modeling for generating globally optimized features: To mitigate pseudo-sequential dependencies that disordered point clouds readily introduce during spatial modeling, the Mamba structure with sequence feature $\tilde{F}$ input performs channel-dimension flipping operations while simultaneously feeding into another independent Mamba structure. Adaptive fusion $F_{m}$ is then achieved through learnable linear mapping to obtain globally optimized features, as shown in Equation (5).

(5) $$F_{m}=Linear\left(\left(\mathrm{Mamba}(\tilde{F})⊕\mathrm{Mamba}\left(Reverse\left(\tilde{F}\right)\right)\right)\right)$$

where $Reverse\left(\cdot \right)$ denotes the channel-wise reversal operation.

Residual semantic-enhanced feature generation: To suppress the interference of spurious sequential dependencies in unordered point clouds, the globally optimized feature $F_{m}$ and the sequential feature $\tilde{F}$ undergo semantic enhancement via layer normalization (LN) and MLP and are superimposed through a residual connection structure to construct the semantic-enhanced feature ${F_{m}}^{\prime }$, as shown in Equation (6).

(6) $${F_{m}}^{\prime }=MLP\left(LN\left(F_{m}+\tilde{F}\right)\right)+\left(F_{m}+\tilde{F}\right)$$

Dynamic local geometric feature aggregation: Relative positions $\Delta x_{i}^{mj}$ are nonlinearly mapped through an encoder composed of 1D Conv, batch normalization (BN), and Swish activation function to obtain geometric features *g*, as shown in Equation (7).

(7) $$g=Conv_{1D}\left(\sigma_{Swish}\left(BN\left(Conv_{1D}\left(\Delta x_{i}^{mj}\right)\right)\right)\right)$$

As the concatenation of the semantically enhanced feature and the geometric feature, the fused feature is obtained through an MLP consisting of a linear layer, a normalization layer, and a Swish activation function, as shown in Equation (8).

(8) $$f=MLP\left(g⊕{F_{m}}^{\prime }\right)$$

To dynamically adjust the importance of each neighbor across to different feature channels, a learnable scaling vector ϒ and translation vector *β* are randomly initialized, and affine transformation is performed with the fused feature *f* to obtain the local geometric aggregated feature $f^{\prime }$, as shown in Equation (9).

(9) $$f^{\prime }=ϒ⊙f+\beta$$

where ⊙ denotes element-wise multiplication.

#### 3.1.3. Anchor-Guided Deformation Parameter Prediction

To generate fine-grained deformation parameters for local point cloud regions while maintaining global geometric topology, an anchor-guided deformation parameter prediction method is proposed. This method consists of three steps: position-aware anchor attention for generating locally enhanced anchor features, context-guided deformation parameter generation, and token-guided mask matrix generation.

Position-aware anchor attention for anchor local enhanced features: The point cloud coordinates X and the corresponding feature map $f^{\prime }$ are subjected to an anchor selection stage. Specifically, farthest point sampling (FPS) [11] is applied on the coordinates X to obtain an index vector $A_{Index}$, where each index in *a* corresponds to a representative point that maximizes the spatial coverage of the shape. Using this predefined index vector, the anchor coordinates and anchor features are gathered as follows:

$$a=\{a_{1},a_{i},a_{M}\},\;F_{aco}=\{F_{aco_{1}},F_{aco_{2}},\ldots ,F_{aco_{M}}\}.$$

where *M* denotes the number of anchors. The anchor coordinates *a* are converted into positional embedding information $E_{pos}$ through a positional encoder [54]. The anchor features $F_{aco}$ are mapped to query (Q), key (K), and value (V) vectors via learnable mapping weights $W_{Q}$, $W_{K}$, $W_{V}$, with positional information embedded, as shown in Equation (10).

(10) $$\begin{array}{l} Q=F_{\mathrm{aco}}W_{Q}+E_{\mathrm{pos}}\\ K=F_{\mathrm{aco}}W_{K}+E_{\mathrm{pos}}\\ V=F_{\mathrm{aco}}W_{v}+E_{\mathrm{pos}} \end{array}$$

The query (Q), key (K), and value (V) vectors construct anchor local enhanced features $F_{atten}$ through standard point attention, as shown in Equation (11).

(11) $$F_{atten}=Softmax\left(\frac{{\mathrm{QK}}^{⊤}}{\sqrt{d}}\right)V$$

where d denotes the dimension of each attention head.

Context-guided deformation parameter generation: To constrain local deformation, the anchor coordinates generate global context features $F_{global}$ through a globally aware MLP, as shown in Equation (12).

(12) $$F_{\mathrm{global}}=MLP(a^{T})$$

as concatenation of the local anchor enhanced features $F_{atten}$ and $F_{global}$ along the channel dimension, they are passed to the deformation parameter prediction head [46] to obtain deformation parameters *P*, as shown in Equation (13).

(13) $$P=MLP\left(F_{atten}⊕F_{global}\right)$$

where ⊕ denotes expanded concatenation.

Token-guided mask generation: To enhance the model’s focus on key structural points, the anchor local representation features $F_{atten}$ are added to the local geometric aggregated features $f^{\prime }$ and then passed through a local mask generation MLP [46] to obtain the local mask $M_{\mathrm{local}}$, as shown in Equation (14).

(14) $$M_{\mathrm{local}}=MLP(F_{\mathrm{atten}}+f^{\prime })$$

The feature $T_{\mathrm{cls}}$ (as shown in Equation (4)) is passed through a global mask attention generation MLP to obtain the global mask $M_{\mathrm{global}}$, as shown in Equation (15).

(15) $$M_{\mathrm{global}}=MLP(T_{\mathrm{cls}})$$

Both generate mask matrix vectors through a Multi-Layer Perceptron (MLP) and the Gumbel–Softmax function [55] to generate the mask matrix vector, as shown in Equation (16).

(16) $$M=GumberlSoftMax\left(MLP\left(M_{\mathrm{local}},M_{\mathrm{global}}\right)\right)$$

### 3.2. Frequency-Domain Adaptive Multi-Scale Dual-Channel Deformation Parameter Prediction

**Figure 3:**
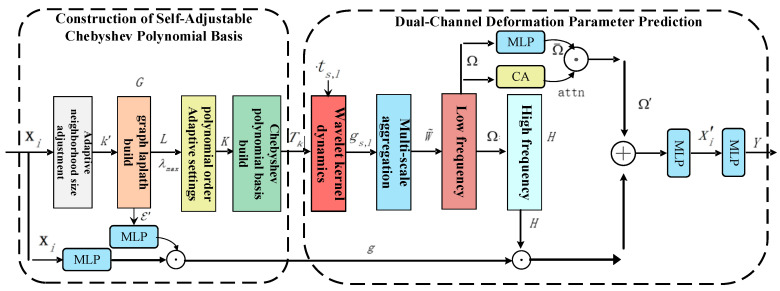
Overview of the frequency-domain adaptive multi-scale dual-channel deformation parameter prediction framework.

Given that point clouds are susceptible to occlusion, missing data, and noise interference during actual acquisition, high-frequency features are often more affected, leading to instability in model outputs. In contrast, low-frequency components of point clouds can better reflect their geometric backbone information and exhibit stronger noise-resistant robustness. Therefore, a frequency-domain adaptive multi-scale dual-channel deformation parameter prediction method is proposed, which mainly consists of two components: the construction of a self-adjustable Chebyshev polynomial basis with adaptive graph and representation and dynamic multi-scale frequency-domain dual-channel deformation parameter prediction, as shown in Figure 3.

#### 3.2.1. Construction of Self-Adjustable Chebyshev Polynomial Basis with Adaptive Graph Representation

Chebyshev polynomial bases are adopted for spectral modeling to avoid explicit eigen decomposition of the graph Laplacian, which is computationally expensive and unstable for dynamically constructed point cloud graphs. However, fixed Chebyshev filters implicitly assume consistent spectral distributions across different point clouds, which is often violated due to variations in sampling density, geometric scale, and noise patterns. To address this limitation, an adaptive frequency-domain feature construction method is proposed, where the polynomial coefficients are learned in a data-driven manner. This design enables the spectral filters to dynamically adjust their frequency responses according to the underlying point cloud structure, allowing for the more flexible suppression of high-frequency noise while preserving discriminative low-frequency geometric information. Specifically, it includes two core steps: the construction of an adaptive graph Laplacian and matrix and the construction of a self-adjustable Chebyshev polynomial basis. The former constructs a graph structure with variable topology through a sparsity-adjusted neighborhood adaptive strategy, providing structural constraints for subsequent spectral decomposition; the latter dynamically determines the polynomial order based on the spectral characteristics of the Laplacian matrix, generating a Chebyshev polynomial basis with frequency self-adjustment capability.

Construction of adaptive graph Laplacian matrix: To adapt to the differences in density and distribution of different point clouds, a sparsity hyperparameter Z∈(0,1] is established and the local neighborhood size $k^{\prime }$ of the point cloud is adaptively adjusted according to the benchmark number of nearest neighbors *k*, as shown in Equation (17).

(17) $$k^{\prime }=\max\left(1,\left\lfloor k\cdot Ζ\right\rfloor \right)$$

By constructing a $k^{\prime }-nearest$ neighbor graph [12] G=(V,E), where the vertex set $V=\{1,2,\ldots ,k^{\prime }\}$ corresponds to the point cloud $x_{i}$ and the edge set E={(i,j)|j∈V} represents local geometric adjacency relationships, the entire set of the edge feature is denoted as $E^{\prime }=\{e_{i,j}|(i,j)\in E\}$. The adjacency graph *G* undergoes standard graph signal processing [22] to transform into the adjacency matrix *A*. The adjacency matrix *A* is then used to construct the graph Laplacian matrix *L* according to the symmetric normalization method, as shown in Equation (18).

(18) $$L=I-D^{-1/2}\cdot \min(A+A^{⊤},1)\cdot D^{-1/2}$$

Self-adjustable Chebyshev polynomial basis construction: To fully capture information at different frequencies within the graph structure and achieve local filtering and multi-scale modeling of the signal, the maximum eigenvalue $\lambda_{max}$ of the Laplacian matrix *L* is dynamically determined by the clamp structure to set the polynomial degree *K*, as shown in Equation (19).

(19) $$K=clamp\left(\left[\lambda_{max}\cdot K\right],2,K\right)$$

The Laplacian matrix *L* and polynomial order *K* are constructed through a recurrence relation [40] to form an adaptive-order Chebyshev polynomial basis ${\left\{T_{k}\right\}}_{k=0}^{K}$, as shown in Equation (20).

(20) $$T_{0}=I,\;T_{1}=L,\;T_{k}=2LT_{k-1}-T_{k-2}\;(k\geq 2)$$

#### 3.2.2. Dynamic Multi-Scale Frequency-Domain Dual-Channel Deformation Parameter Prediction

To achieve efficient geometric representation and controllable deformation prediction of 3D point clouds in the frequency domain, a dynamic multi-scale frequency-domain dual-channel deformation parameter prediction method is designed. This method aims to learn optimal wavelet filter kernels and frequency response patterns in an end-to-end manner, thereby realizing fine-grained modeling of deformation-sensitive features at the multi-scale structural level of point clouds. It mainly consists of three steps: construction of dynamic learnable wavelet kernels, multi-scale frequency-domain feature aggregation, and dual-channel frequency-domain feature deformation prediction.

Construction of dynamic learnable wavelet kernels: To enable the learnability of wavelet filtering and end-to-end optimization capability, a scale parameter $t_{s,l}\in R^{+}$ is designed, and a parameterized wavelet kernel $g_{s,l}\left(K\right)$ is obtained through an exponential decay function, as shown in Equation (21).

(21) $$g_{s,l}\left(K\right)=\exp(-t_{s,l}\cdot K)$$

Multi-scale frequency-domain feature aggregation: To accurately extract response features of input signals in different frequency dimensions and realize comprehensive modeling of multi-structural granularity information in point cloud data, wavelet coefficients at a specific scale *S* are calculated using the wavelet kernel $g_{s,l}\left(K\right)$ and weighted Chebyshev basis $T_{k}$. Wavelet coefficients at various scales are aggregated to generate multi-scale features $\tilde{W}$, as shown in Equation (22).

(22) $$\tilde{W}=\frac{1}{S}\sum_{s=1}^{S}\left(\sum_{k=0}^{K}g_{s,l}\left(K\right)T_{k}x_{i}\right)$$

Dual-channel frequency-domain feature deformation prediction: To enable subsequent models to perform differentiated modeling for low-frequency and high-frequency semantic features of point clouds, wavelet coefficients are hierarchically parsed. Among the multi-scale features $\tilde{W}$, the representation of the S-th layer (coarsest scale) is used as the low-frequency component $\Omega \in R^{B\times N\times C}$ encoding the point cloud, as shown in Equation (23).

(23) $$\Omega =\tilde{W}[:,0,:,:]$$

Among the multi-scale features $\tilde{W}$, the wavelet coefficients from the 1st to the (S-1)-th layers are averaged and aggregated along the hierarchical dimension as the high-frequency component $H\in R^{B\times N\times C}$ of the 3D point cloud, as shown in Equation (24).

(24) $$H=\frac{1}{K-2}\sum_{k=1}^{K-1}\tilde{W}[:,S-1,:,:]$$

To enhance the expression of low-frequency information of 3D point clouds and suppress high-frequency noise interference, the low-frequency component Ω is input into an MLP composed of fully connected layers, batch normalization (BN) layers, and LeakyReLU activation function to obtain the component $\bar{\Omega }$, as shown in Equation (25).

(25) $$\bar{\Omega }=MLP\left(\Omega \right)$$

To adjust the importance of features in different channels, $\bar{\Omega }$ constructs weight parameters attn through a channel attention (CA) mechanism [56], as shown in Equation (26).

(26) $$\mathrm{attn}=CA\left(\bar{\Omega }\right)$$

where $CA\left(\cdot \right)$ denotes the channel attention mechanism.

The component $\bar{\Omega }$ is multiplied by the weight parameters attn in the element-wise manner and then combined with the low-frequency component through residual connection to obtain the frequency-domain enhanced component $\Omega^{\prime }$, as shown in Equation (27).

(27) $$\Omega^{\prime }=\Omega +\mathrm{attn}⊙\bar{\Omega }$$

To remove structural noise in high-frequency information and prevent augmented samples from being disturbed, edge features and point cloud $x_{i}$ are, respectively, mapped through MLPs. The mapping results are multiplied in the element-wise manner to obtain the modulation coefficient *g*, as shown in Equation (28).

(28) $$g=MLP\left(X\right)⊙MLP(E^{\prime })$$

where ⊙ denotes element-wise multiplication.

The modulation coefficient *g* is used to adjust the high-frequency component *H*, and the product of the two is concatenated with the low-frequency enhanced component $\Omega^{\prime }$ along the channel dimension. The concatenated features undergo feature projection through an MLP to obtain the enhanced point spatial representation ${x^{\prime }}_{i}$, as shown in Equation (29).

(29) $${x^{\prime }}_{i}=MLP\left(\Omega^{\prime }⊕\left(H⊙g\right)\right)$$

The enhanced point spatial representation ${x^{\prime }}_{i}$ is fed into an MLP-based prediction head to generate deformation parameters *Y*, as shown in Equation (30).

(30) $$Y=MLP({x^{\prime }}_{i})$$

### 3.3. Spatial–Frequency Domain Deformation Feature Adaptive Fusion Modeling

#### 3.3.1. Fusion-Based Adversarial Sample Generation

**Figure 4:**
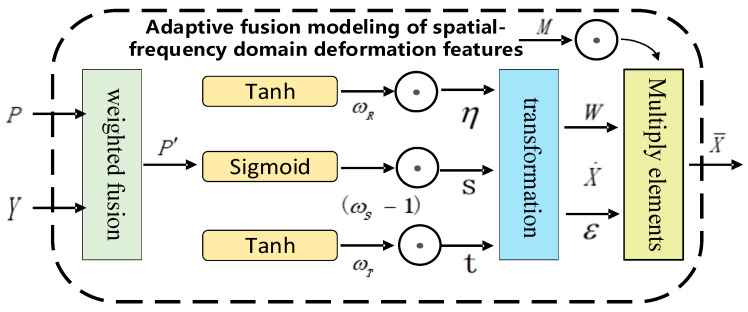
Overview of the spatial–frequency domain deformation feature adaptive fusion modeling framework.

To enhance the diversity and structural consistency of point cloud augmentation while ensuring its challenge for downstream tasks, a spatial–frequency domain deformation feature adaptive fusion modeling method is proposed, as shown in Figure 4. This method enables complementary modeling of multi-modal augmented features between the spatial domain and frequency domain and adaptively integrates the two types of deformation parameters through learnable fusion coefficients to improve the diversity of the point cloud samples.

Specifically, the local deformation parameters *P* output by the spatial-domain adaptive bidirectional Mamba deformation parameter prediction method and the deformation parameters *Y* output by the frequency-domain adaptive multi-scale dual-channel deformation parameter prediction method are weighted and fused according to predefined weights *α* and 1−α to obtain the fused deformation parameters $P^{\prime }$, as shown in Equation (31).

(31) $$P^{\prime }=\alpha P+(1-\alpha )Y$$

$P^{\prime }$ achieves nonlinear mapping through the hyperbolic tangent function (tanh) and Sigmoid function to obtain the rotation vector *η* scaling vector ***s***, and rotation vector **t**, respectively. The offset between the original point cloud $x_{i}$ and the corresponding anchor points $a_{i}$ undergoes rotation, scaling, and translation operations in sequence to obtain the local deformation result, which is then multiplied element-wise by the mask matrix *M* obtained from Equation (16) to generate the augmented point cloud sample ${\bar{x}}_{i}$, as shown in Equation (32).

(32) $${\bar{x}}_{i}=\frac{\sum_{i}w\left(\left(\eta s\right)\left(x_{i}-a_{i}\right)+t\right)}{\sum_{i}w+\varepsilon }⊙M$$

where ε is a small constant to avoid division by zero.

#### 3.3.2. Adversarial Loss Computation

To ensure that the learned spatial–frequency domain deformations possess diversity, authenticity, and discriminability, an adversarial learning framework is constructed between the deformation generator *G* and the point cloud discriminator *D*. The working mechanism of the generator has been described in the previous section, producing fused deformation parameters and the final augmented point cloud $\bar{X}$ (Equations (30) and (31)). This section details the architecture of the discriminator, its adversarial loss function, and the classification-aware feedback mechanism.

The discriminator *D* maps an input point cloud $X\in R^{N\times 3}$ to a scalar probability, indicating the likelihood that **X** is a real sample. The input is reshaped to match $\left[B,N,3\right]$ and processed through a set abstraction module with spectral-normalized 1 × 1 convolutions and global pooling to extract a global feature **F**, as shown in Equation (33).

(33) F=SA(X)

where SA(·) represents the set abstraction layer integrated with spectral normalization 1 × 1 convolution and max pooling, and $F\in R^{1024}$ is the global shape feature vector extracted. This feature **F** is mapped through a stack of spectral-normalized fully connected layers with LeakyReLU activation to obtain a latent embedding **z**, which is finally projected to a scalar probability via a Sigmoid function, as shown in Equation (34).

(34) $$z=\Phi_{\mathrm{SN}\text{-}\mathrm{FC}}(F)$$

where $\Phi_{\mathrm{SN}\text{-}\mathrm{FC}}(\cdot )$ represents the composite function of multiple fully connected layers with spectral normalization.

The generator *G* is trained to produce augmented point clouds that fool the discriminator. Its adversarial loss $L_{\mathrm{GAN}}^{G}$ forces the generated samples to approximate the real point cloud distribution in terms of the discriminator’s output, as shown in Equation (35).

(35) $$L_{\mathrm{GAN}}^{G}=L_{\mathrm{BCE}}(D(\bar{X}),y_{\mathrm{real}})$$

The discriminator is optimized using a label-smoothed binary cross-entropy loss to distinguish real point clouds $X_{\mathrm{real}}$ from generated samples $\bar{X}=D(X_{real})$, as shown in Equation (36).

(36) $$L_{D}=\frac{1}{2}\left[L_{\mathrm{BCE}}(D(X_{\mathrm{real}}),y_{\mathrm{real}})+L_{\mathrm{BCE}}(D(\bar{X}),y_{\mathrm{fake}})\right]$$

where $L_{BCE}(\cdot ,\cdot )$ denotes the binary cross-entropy loss function, and $y_{\mathrm{real}}\approx 0.9$ and $y_{\mathrm{fake}}\approx 0.1$ are the smoothed labels for real and generated samples, respectively. To ensure that the generated samples are not only realistic but also challenging for downstream classification tasks, a classification-aware feedback mechanism is introduced. Let *f* denote the downstream classifier and $L_{cls}(f(X),y)$ represent the classification loss on point cloud **X** with label **y**. The classification losses for real and generated samples are as follows:

$$L_{\mathrm{real}}=L_{\mathrm{cls}}(f(X_{\mathrm{real}}),y),L_{\mathrm{fake}}=L_{\mathrm{cls}}(f(\bar{X}),y)$$

A dynamic difficulty coefficient $\rho_{e}$ is defined to progressively increase the challenge of generating samples during training, as shown in Equation (37).

(37) $$\rho_{e}=\rho_{\mathrm{start}}+\frac{e}{E_{\max}}\left(\rho_{\mathrm{end}}-\rho_{\mathrm{start}}\right)$$

where *e* is the current training epoch, $E_{\max}$ is the total number of epochs, and $\rho_{\mathrm{start}}$ and $\rho_{\mathrm{end}}$ are preset hyperparameters.

The adversarial feedback loss is then computed to enforce the generated sample classification loss to scale proportionally with the real sample loss, as shown in Equation (38).

(38) $$L_{\mathrm{fb}}=\left|1-\exp\left(L_{\mathrm{fake}}-\rho_{e}L_{\mathrm{real}}\right)\right|$$

Finally, the generator minimizes the combination of the adversarial loss and the feedback loss to balance realism and task-aware difficulty, as shown in Equation (39).

(39) $$L_{G}=L_{\mathrm{GAN}}^{G}+\lambda_{\mathrm{fb}}L_{\mathrm{fb}}$$

where $\lambda_{fb}$ is a weighting factor controlling the relative importance of the feedback loss. The discriminator and generator are trained alternately to form a closed-loop adversarial feedback system, in which the discriminator supervises the realism of generated samples and the feedback mechanism guides the generator to produce task-challenging yet learnable point cloud augmentations.

## 4. Experimental Results and Analysis

### 4.1. Experimental Datasets

To evaluate the robustness of the proposed 3D point cloud enhancement method in real-world scenarios and its generalization capability under various types of perturbations, this study employs two major mainstream point cloud damage benchmark datasets, namely ModelNet40-C [42] and ScanObjectNN-C [34], for experimentation. These datasets exhibit significant complementarity in data sources, scene complexity, and noise patterns, comprehensively covering application scenarios ranging from controlled synthetic damage to real-world environmental noise. This provides robust assurance for the comprehensiveness, reliability, and practical value of the evaluation results. ModelNet40-C is constructed from the clean ModelNet40 CAD point cloud, generating controlled damage versions by injecting eight types of structured perturbations: scaling (Sca) simulates dimensional inconsistencies caused by changes in line-of-sight or viewing angle; point cloud jitter (Jit) simulates sensor measurement errors by adding Gaussian random noise; global point deletion (D-G) simulates large-scale scanning gaps or occlusions; local point deletion (D-L) simulates partial structural damage or local occlusions; global point addition (A-G) simulates background scattering or overall noise introduction; local point addition (A-L) simulates noise clustering or high-reflectivity artifacts in specific regions; random rotation (Rot) tests model pose invariance; and mixed contamination error (Mce) comprehensively evaluates overall model robustness under multi-category damage superposition. This dataset features clearly defined disturbance types and controllable parameters, enabling detailed analysis of model stability when addressing structural damage under ideal point cloud conditions. Another referenced dataset, ScanObjectNN-C, more closely mirrors real-world point cloud damage scenarios. Its raw point clouds originate from the most challenging PB-T50-RS variant test set within ScanObjectNN. This variant was directly captured from actual indoor scanning environments, inherently incorporating complex real-world factors such as cluttered backgrounds, partial viewpoint loss, sensor noise, irregular point cloud density, and occlusion artifacts. It further introduces random translations, rotations, and simulated occlusions on top of the original scans, establishing a highly demanding benchmark for evaluating models’ real-world generalization capabilities. ScanObjectNN-C [34] processes the original point cloud through seven damage types (“Jitter”, “Drop Global/Local”, “Add Global/Local”, “Scale”, “Rotate”) and five severity levels, enabling a comprehensive examination of model robustness in real-world scenarios. Notably, unlike ModelNet40-C derived from CAD models, all ScanObjectNN-C samples originate from real-world scans, making its damage effects more representative of actual application conditions. In summary, this study comprehensively evaluates the performance of the augmentation strategy across different scenarios by conducting experiments on ModelNet40-C (controlled geometric damage scenarios) and ScanObjectNN-C (real-world noise scenarios). This provides a thorough and reliable basis for assessing the robustness and generalization capabilities of the proposed method.

### 4.2. Experimental Details

The proposed method in this paper is implemented on the open-source deep learning framework PyTorch (version 2.4.1). All experiments are conducted on a single hardware setup configured as follows: an NVIDIA GeForce RTX 4070/PCIe/SSE2 graphics card (12 GB VRAM), a 12th Gen Intel^®^ Core™ i7-12700 CPU (20 cores), and 64GB RAM. The operating system is Ubuntu 20.04 LTS, with PyTorch version 2.4.1 and CUDA version 11.8.

The model is trained end-to-end for 300 epochs on the ModelNet40-C and ScanObjectNN-C datasets, with the Adam optimizer configured to an initial learning rate of 0.001 and a batch size of 32. To enhance the model’s generalization capability and robustness, a suite of data augmentation strategies is employed during training, including random rotation, scaling, jittering, and point cloud dropout. For the sake of fair comparison, all competing methods share the identical network backbone and training pipeline. To standardize the severity settings of diverse corruption types, DGCNN is selected as the baseline model. Following the point cloud corruption evaluation framework proposed by Ren et al. (2022) [57], the mean corruption error (mCE) is adopted as the primary evaluation metric. Its core principle lies in normalizing performance differences between the proposed model and the baseline across five corruption levels for each corruption type (i), yielding the corruption error (CE), as defined in Equation (40).

(40) $$CE_{i}=\frac{\sum_{l=1}^{5}\left(1-OA_{i,l}\right)}{\sum_{l=1}^{5}\left(1-OA_{i,l}^{\mathrm{DGCNN}}\right)}$$

where $OA_{i,l}$ denotes the overall accuracy of the proposed model under corruption type (i) and severity level (l), while $OA_{i,l}^{\mathrm{DGCNN}}$ represents the corresponding accuracy of the DGCNN baseline. The mCE is then calculated as the average of CE across all *N* = 7 corruption types, as shown in Equation (41):

(41) $$mCE=\frac{1}{N}\sum_{i=1}^{N}CE_{i}$$

### 4.3. Experimental Results

#### 4.3.1. Robustness Comparison on Point Cloud Classification

To comprehensively evaluate the effectiveness of the proposed SFD-ADNet, we conduct robustness experiments from both global recognition and local dense prediction perspectives. Specifically, we assess classification robustness under diverse point cloud corruptions on ModelNet40-C and ScanObjectNN-C and further examine whether the learned adaptive deformation preserves fine-grained geometric consistency through part segmentation experiments on the ShapeNet Part dataset. This unified evaluation protocol allows us to analyze not only the robustness gains under severe structural damage but also the impact of the proposed spatial–frequency adaptive deformation on local shape integrity.

To validate the robustness and effectiveness of the proposed 3D point cloud data augmentation method in enhancing the structural damage detection performance of models under ideal conditions with high-quality clean point clouds, we conducted comparative experiments on the ModelNet40-C [57] standard dataset. The experiments evaluated the proposed enhancement method in conjunction with 10 classical baseline models and 5 mainstream enhancement methods. Performance of the enhanced models was quantified across eight key metrics (lower metric values indicate stronger robustness under corresponding perturbation scenarios), with the results shown in Table 1. On the PointNet++ [12] backbone, SFD-ADNet achieves error rates of 85.6% and 99.8% under scaling (Sca) and jitter (Jit) perturbations, significantly reducing the original model’s 87.2% and 117.7% rates. Its performance surpasses mainstream augmentation methods like PointWOLF [58] and RSMix [59], demonstrating its effective suppression of high-frequency noise perturbations. Under structurally destructive perturbations such as global point deletion (D-G) and local point deletion (D-L), SFD-ADNet achieved errors of 54.9% and 61.3%, respectively, exhibiting stable overall performance. In point cloud density perturbation scenarios involving global point addition (A-G) and local point addition (A-L), its errors were 32.4% and 30.6%, respectively, maintaining its performance advantage over baseline models. 

On the more robust PointNeXt [66] backbone, SFD-ADNet achieves an exceptionally low mean classification error (mCE) of 67.2%, representing a reduction of over 21% compared to the original PointNet++ (107.2%) and PointNeXt [66] (85.6%). Under typical perturbation scenarios such as jitter (Jit: 99.8%), global point deletion (D-G: 54.9%), and local point deletion (D-L: 61.3%), SFD-ADNet’s error performance significantly outperformed PointWOLF [58], Wolfmix [57], and other enhancement strategies. Under rotational disturbance (Rot), the error is substantially reduced to 58.3%, representing an improvement of over 60% compared to the original model’s 146.0%, achieving the current state of the art in pose robustness. In summary, SFD-ADNet demonstrates comprehensive robustness against scaling transformations, noise perturbations, structural missingness, and pose variations across both backbone architectures. This validates its joint air-frequency domain modeling strategy’s strong generalization and robustness under diverse structural damage conditions. This advantage stems from the proposed adaptive bidirectional Mamba local geometric feature encoder: by modeling long-range dependencies, it captures global structure and contextual information within point clouds, effectively mitigating perturbations such as scaling (Sca), rotation (Rot), and global density variations (D-G) and simultaneously employing a local geometric aggregation mechanism to adaptively weight and fuse neighborhood features. This approach integrates multi-source information while preserving relative point positions, enabling stable feature extraction even under local perturbations like jitter (Jit), local point deletion (D-L), and local point addition (A-L). The extracted features feed into an adaptive deformation parameter prediction network, enabling the generation of robust and diverse point cloud samples. This significantly enhances the robustness of downstream classification tasks.

To validate that the 3D point cloud data augmentation method proposed in this paper can enhance the model’s robustness and generalization against structural damage in realistic complex scenarios, comparative experiments were conducted on the ScanObjectNN-C standard dataset, covering three mainstream backbone networks: DGCNN [22], PointNet++ [12], and PointNeXt [66]. Performance evaluation was performed across seven typical point cloud perturbation types (scaling (Sca), jittering (Jit), global point deletion (D-G), local point deletion (D-L), global point addition (A-G), local point addition (A-L), rotation (Rot)), measuring overall accuracy (OA, higher is better), and mean classification error (mCE, lower is better; quantifying the average performance degradation across perturbations), as shown in Table 2. On the DGCNN [22] backbone, the proposed SFD-ADNet achieves significant performance improvements: the OA under clean settings reaches 90.8%, exceeding the baseline DGCNN (85.8%) by 5.0 percentage points; the mCE decreases to 75.6%, outperforming methods like RSMix [59] (96.9%) and PointWOLF [58] (99.6%) by a wide margin. In structurally destructive corruptions, SFD-ADNet achieves notable error reductions, namely Sca (49.2), Jit (109.0), D-G (68.5), A-G (64.1), and A-L (83.5), demonstrating clear advantages over the baseline, whose CE values are 100.0 across all perturbations. On PointNet++ [12], SFD-ADNet consistently enhances robustness: the OA rises to 89.1% (vs. baseline 86.2%), and the mCE drops to 76.4% (vs. baseline 96.9%), surpassing PointWOLF [48] (96.4%), Wolfmix [57] (87.8), and RSMix [59] (91.9) in corruption resistance. Improvements are prominent in scenarios like D-G (47.2) and D-L (58.1), highlighting the method’s ability to maintain geometric topology even when point cloud structure is disturbed. On the high-performance PointNeXt [51] architecture, SFD-ADNet exhibits the most prominent advantages: its OA reaches 99.4% (the highest among all tested configurations vs. baseline 87.3%), and the mCE decreases to 69.5% (vs. baseline 92.1%), outperforming mainstream augmentation approaches such as RSMix [59] (88.2%) and Wolfmix [57] (86.9%). The method maintains robustness under fine-grained perturbations such as Jit (86.9) and coarse structural degradation such as D-G (55.9). For rotation corruption, SFD-ADNet reduces the CE to 39.3%, a dramatic improvement over the baseline 99.5%. Overall, SFD-ADNet demonstrates strong performance on both clean point clouds and the challenging ScanObjectNN-C benchmark (with multiple real-world disturbances). It maintains high accuracy even when point clouds undergo substantial structural corruption, benefiting from the integration of spatial-domain adaptive bidirectional Mamba-based deformation parameter prediction and frequency-domain adaptive multi-scale dual-channel deformation modeling. These components jointly suppress noisy geometric relationships, amplify informative structural cues, and guide the generator to produce diverse, structurally coherent augmented samples, thereby enhancing model robustness and generalization via increased data diversity.

#### 4.3.2. Training Cost, Scalability, Deployment, and Overfitting Analysis

Table 3 reports the training-time cost of PointNeXt equipped with two different training-stage data augmentation strategies, namely AdaptPoint and the proposed SFD-ADNet, where the reported training time is averaged over all mini-batches within each epoch. When replacing AdaptPoint with SFD-ADNet, the training time increases from 3.8 min to 7.2 min per epoch, primarily due to the adaptive deformation modeling performed jointly in the spatial and frequency domains. Compared with conventional lightweight augmentation strategies (e.g., random jittering or scaling) and simpler pretraining-based methods, SFD-ADNet intentionally introduces higher training-time cost by explicitly learning deformation parameters conditioned on point cloud features rather than applying handcrafted or heuristic perturbations. In essence, the proposed augmentation framework trades additional training time for improved data quality and robustness, enabling the generation of augmented point clouds that exhibit stronger resistance to structural corruption while better preserving critical geometric features. Importantly, this extra computation is incurred only during the training stage, and as demonstrated in Table 2, these high-quality augmented samples provide more discriminative supervision for downstream models, leading to substantial improvements in robustness and generalization across diverse structural perturbations.

In terms of memory consumption, all experiments were conducted on a single NVIDIA RTX 3070 GPU with 12 GB memory. Despite the additional components introduced by SFD-ADNet, including adaptive deformation parameter prediction and multi-scale frequency-domain modeling, the peak GPU memory usage increases moderately from 9.6 GB (AdaptPoint) to 10.8 GB (SFD-ADNet), remaining well within the capacity of commonly available hardware. From a scalability perspective, SFD-ADNet operates on local and multi-scale feature representations rather than global point-wise pairwise interactions, avoiding quadratic complexity with respect to the number of points. Consequently, its computational cost scales approximately linearly with point cloud size and batch size, and the augmentation module can be seamlessly integrated into PointNeXt as well as other mainstream backbones such as DGCNN and PointNet++, without modifying inference architectures. Since both AdaptPoint and SFD-ADNet are applied exclusively during training, and SFD-ADNet introduces no additional parameters, computation, or memory overhead during inference, the trained PointNeXt models retain identical inference speed, model size, and latency, making them fully compatible with real-time and resource-constrained deployment scenarios.

Despite incorporating multiple fusion strategies, the overall number of learnable parameters in SFD-ADNet remains moderate due to the use of polynomial spectral approximation and lightweight state-space modeling. Combined with the moderate yet standard training datasets—ModelNet40 (9843 training/2468 test samples) and ScanObjectNN (~15,000 instances across 15 categories)—the model does not exhibit overfitting. This is further supported by the test set performance and the robustness evaluations on ModelNet40-C and ScanObjectNN-C, where the method maintains strong accuracy under diverse structural corruptions. These observations indicate that the proposed augmentation framework enhances feature learning and generalization rather than memorizing the training data, confirming the reliability of its performance even on moderately sized datasets.

#### 4.3.3. Robustness and Generalization on Point Cloud Part Segmentation

Table 4 reports part segmentation results on the ShapeNet Part benchmark. When integrated with PointNet++ and DGCNN, SFD-ADNet consistently improves mean IoU across most object categories. Specifically, on the PointNet++ [12] backbone, SFD-ADNet improves the overall mIoU from 82.3% to 84.7%, yielding a +2.4% absolute gain, with notable improvements on structurally complex categories such as Car, Chair, Motorbike, and Rocket. On DGCNN, the proposed method further increases mIoU from 82.6% to 85.4%, corresponding to a +2.8% improvement, while achieving consistent gains across nearly all part categories. These results indicate that the proposed spatial–frequency adaptive deformation does not introduce destructive local distortions but instead preserves part-level geometric coherence while enhancing feature robustness. This demonstrates that SFD-ADNet generalizes effectively beyond global classification to dense local prediction tasks.

#### 4.3.4. Experimental Results of Point Cloud Attack Defense

This experiment aims to verify that SFD-ADNet outperforms other point cloud defense methods in resisting unnatural, extreme, and unpredictable perturbations, achieving both “defense” and augmentation effects. We evaluate the performance of the trained PointNet++ against malicious perturbation attacks, including point perturbation, standalone point addition attacks (Add-CD and Add-HD), kNN-based attacks, and point deletion attacks of varying intensities (Drop-100 and Drop-200) in comparison with mainstream defense methods. The backbone network and training strategies are consistent with baseline methods to ensure fair comparison, as shown in Table 5. On the overall perturbation resilience metric Perturb, SFD-ADNet achieved a score of 88.49%, surpassing all comparison methods including IF-Defense and AdaptPoint, demonstrating its strong robustness in global structural recovery. In point addition attacks, SFD-ADNet achieved the highest scores of 81.76% and 77.52% on Add-CD and ADD-HD, respectively, significantly outperforming classical methods such as DUP-Net, SOR, and IF-Defense. This demonstrates its superior ability to maintain geometric consistency when resisting point addition perturbations. Under KNN adversarial perturbations, SFD-ADNet also achieved the best performance at 87.16%, further validating its robustness against local neighborhood structure disruptions. Regarding point deletion attacks, SFD-ADNet achieved 85.92% under Drop-100, closely matching the top method AdaptPoint’s 86.55. In the more severe Drop-200 scenario, it scored 80.61%, maintaining an advantage over most traditional methods. This outstanding performance stems primarily from SFD-ADNet’s designed frequency-domain adaptive multi-scale dual-channel deformation parameter prediction method based on graph wavelet transform. This approach extracts and enhances low-frequency features in point clouds, thereby stably capturing object skeleton information and structural consistency. This mechanism provides more reliable structural constraints for subsequent geometric transformations, effectively mitigating interference from high-frequency noise. Consequently, high classification accuracy is maintained even when encountering various point cloud attacks.

### 4.4. Ablation Experiments

#### 4.4.1. Ablation Experiment of the Bidirectional Semantic-Aware and Geometry-Adaptive Encoder

Ablation experiments were conducted to assess the impact of the bidirectional sequence modeling (Bi-SSM) and local geometric aggregation (LGA) modules in the Mamba3D-enhanced semantic encoder. As shown in Table 6, removing the LGA module increases the mean corruption error (mCE) from 69.5% to 72.5%, highlighting the importance of local structural reasoning. Replacing Bi-SSM with unidirectional sequence modeling (SSM) raises the mCE to 73.2%, indicating that bidirectional modeling better captures global context and enhances robustness. This study also explored other sequence modeling configurations: the mCE for Tri-SSM was 70.6%, while that for One-SSM was 71.3%, both inferior to the Bi-SSM design. These results confirm that our approach of combining Bi-SSM with LGA achieves optimal robustness, reducing mCE by up to 3.7% compared to unidirectional sequence modeling.

#### 4.4.2. Ablation of Deformation, Mask, and LFDG

This study evaluates the contributions of three key components: the anchor-based deformation network (deformation), the structure-aware mask network (Mask), and the dynamic multi-scale frequency-domain dual-channel deformation parameter prediction (DCDPP). As shown in Table 7, adding each component individually to the baseline reduces the mean corruption error (mCE) from 92.1 to 76.4, 77.1, and 78.2, respectively. Combining all three achieves the best result, lowering the mCE to 69.5. This significant improvement highlights the synergy between spatial deformation, structural masking, and low-frequency feature learning, which together enhance robustness by suppressing high-frequency noise while preserving global geometric structure.

Notably, although each component independently reduces mCE by more than 13%, their combination yields an additional performance gain beyond linear accumulation. This indicates a strong synergistic effect between spatial deformation, structural masking, and frequency-domain low-frequency guidance, where spatial modules improve geometric alignment while spectral modeling stabilizes deformation under structural corruption. Taken together, the ablation results in Section 4.4.1 and Section 4.4.2 indicate that robustness gains arise from the complementary interaction between spatial-domain sequential modeling and frequency-domain deformation learning. While the bidirectional Mamba encoder primarily stabilizes global and local geometric dependencies, the frequency-domain module further regularizes deformation by suppressing high-frequency noise. Neither component alone achieves optimal robustness, highlighting the necessity of joint spatial–frequency modeling.

#### 4.4.3. Sensitivity Analysis of Anchor Number and Loss Weight

To evaluate the sensitivity and robustness of SFD-ADNet with respect to key hyperparameters, we conduct a systematic ablation study on the number of anchor points and the loss weight λ, as summarized in Table 8. Regarding the number of anchors, the proposed model achieves optimal robustness when four anchors are used, yielding an mCE of 69.5%. When the anchor number is reduced to two, the mCE moderately increases to 71.4%, indicating limited local deformation expressiveness. Increasing the anchor numbers to eight and sixteen results in mCE values of 72.6% and 74.3%, respectively, suggesting that excessive anchor density may disrupt global geometric consistency. Notably, within the reasonable range of 2–8 anchors, performance degradation remains gradual, demonstrating that the model is not overly sensitive to the exact anchor configuration. A similar trend is observed for the loss weight λ. The best performance is obtained at λ = 1 (mCE = 69.5%). When λ is set to 0.5, the mCE slightly increases to 70.4%, indicating insufficient regularization. Increasing λ to 2 and 4 leads to mCE values of 71.1% and 73.2%, respectively, suggesting that overly strong constraints may impair feature alignment. Importantly, moderate variations around the optimal setting result in only marginal performance changes.

Overall, these results demonstrate that SFD-ADNet maintains stable performance across a wide range of hyperparameter values. Performance degradation occurs mainly under extreme configurations, confirming that the proposed framework exhibits strong robustness and low sensitivity to moderate hyperparameter variations.

## 5. Conclusions

This paper proposes SFD-ADNet, a spatio-frequency dual-domain adaptive deformation framework for three-dimensional point cloud data augmentation, designed to improve robustness and generalization under natural corruptions and adversarial perturbations. Extensive experiments on the ModelNet40-C and ScanObjectNN-C benchmarks, conducted with three representative backbone networks—DGCNN, PointNet++, and PointNeXt—demonstrate that SFD-ADNet consistently improves classification accuracy while significantly reducing the mean corruption error across diverse structural degradations, including scaling, jittering, point deletion/addition, and rotation. Additional adversarial robustness evaluations show that SFD-ADNet outperforms existing defense-oriented augmentation methods under point addition, deletion, and neighborhood-based attacks, confirming its effectiveness as both a data augmentation and robustness enhancement strategy. The performance gains arise from complementary spatio-frequency modeling: in the spatial domain, a bidirectional Mamba-based adaptive sequential encoder captures long-range dependencies and local structural variations for stable deformation prediction; in the frequency domain, an adaptive multi-scale dual-channel mechanism emphasizes low-frequency structural components while suppressing high-frequency noise, thereby preserving geometric consistency under severe perturbations. By adaptively fusing spatial and spectral deformation parameters, SFD-ADNet achieves a favorable balance between structural fidelity and sample diversity, which is essential for robust downstream learning. Future work will extend SFD-ADNet to dense point cloud understanding tasks, such as semantic segmentation, and explore its integration with robust or self-supervised pretraining paradigms, as well as its applicability to more complex scenarios, including LiDAR point clouds.

## Figures and Tables

**Table 1:** Comparison of mCE (%) performance metrics on the ModelNet40-C dataset.

Method	mCE ↓	Sca ↓	Jit ↓	D-G ↓	D-L ↓	A-G ↓	A-L ↓	Rot ↓
DGCNN [22,46]	100.0	100.0	100.0	100.0	100.0	100.0	100.0	100.0
PointNet [11,46]	142.2	126.6	64.2	50.0	107.2	298.0	159.3	190.2
RSCNN [57,60]	113.0	107.4	117.1	80.6	151.7	71.2	115.3	147.9
SimpleView [57,61]	104.7	87.2	71.5	124.2	135.7	98.3	84.4	131.6
GDANet [57,62]	89.2	83.0	83.9	79.4	102.4	134.6	100.0	80.9
CurveNet [57,63]	92.7	87.2	72.5	71.0	102.4	134.6	100.0	80.9
PAConv [57,64]	110.4	90.4	146.5	100.0	100.5	108.5	129.8	96.7
SMCNet [65]	85.8	100.1	70.7	79.1	69.8	79.7	93.3	79.7
PointNet++ [12,57]	107.2	87.2	117.7	64.1	180.2	61.4	99.3	140.5
+PointWOLF [58]	82.5	81.9	135.1	67.3	130.4	43.1	68.4	51.2
+RSMix [46,59]	86.3	89.4	164.9	48.4	73.9	26.1	32.7	168.4
+Wolfmix [57]	64.1	94.7	137.0	46.0	61.4	29.8	29.5	50.7
+AdaptPoint [46]	63.7	109.6	102.2	36.7	66.7	30.5	40.0	60.5
+SFD-ADNet (our)	84.5	78.9	96.9	55.0	82.0	54.9	85.7	94.3
PointNeXt [57,66]	85.6	90.4	129.7	84.7	95.7	25.1	27.6	146.0
+PointWOLF [58]	79.5	88.3	144.9	113.7	103.9	26.4	28.0	51.2
+RSMix [46,59]	87.9	100.0	159.2	86.7	54.1	23.1	26.5	165.6
+Wolfmix [57]	74.0	84.0	156.0	119.4	56.5	23.7	25.1	56.0
+AdaptPoint [46]	71.1	108.7	89.2	65.0	74.3	34.8	31.7	69.0
+SFD-ADNet (our)	67.2	85.6	99.8	54.9	61.3	32.4	30.6	58.3

Note: “↓” indicates that lower values correspond to better performance.

**Table 2:** Comparison of mCE (%) performance metrics on the ScanObjectNN-C dataset.

Method	OA ↑	mCE ↓	Sca ↓	Jit ↓	D-G ↓	D-L ↓	A-G ↓	A-L ↓	Rot ↓
DGCNN [22,57]	85.8	100.0	100.0	100.0	100.0	100.0	100.0	100.0	100.0
+PointWOLF [46,58]	85.6	99.6	89.5	104.6	104.1	98.3	98.0	100.9	101.6
+RSMix [46,59]	86.5	96.9	103.1	97.4	91.2	85.3	104.8	98.7	97.6
+Wolfmix [57]	87.2	92.3	92.1	102.6	92.6	85.3	96.2	85.7	91.5
+AdaptPoint [46]	84.4	90.2	90.6	107.5	72.3	73.0	93.3	93.3	101.4
+SFD-ADNet (our)	90.8	75.6	49.2	109.0	68.5	91.4	64.1	63.4	83.5
PointNet++ [12,57]	86.2	96.9	89.7	110.3	55.0	127.7	94.7	90.5	110.7
+PointWOLF [58]	86.6	96.4	84.0	108.7	70.5	156.6	87.7	90.9	76.1
+RSMix [46,59]	87.3	91.9	89.0	100.7	55.6	99.0	94.6	89.1	114.9
+Wolfmix [57]	87.5	87.8	79.6	109.0	64.2	117.7	88.1	79.8	76.2
+AdaptPoint [46]	86.3	85.8	89.8	105.7	56.1	69.3	99.8	91.6	88.0
+SFD-ADNet (our)	89.1	76.4	81.2	90.8	47.2	58.1	84.7	78.1	74.3
PointNeXt [12,57]	87.3	92.1	80.3	107.9	80.7	94.2	94.4	87.5	99.5
+PointWOLF [58]	87.4	89.5	81.4	112.9	89.8	92.3	95.0	83.7	71.1
+RSMix [46,59]	88.1	88.2	83.9	107.3	74.9	73.3	96.2	82.9	99.1
+Wolfmix [57]	87.7	86.9	81.9	119.3	89.7	78.0	89.3	89.0	70.0
+AdaptPoint [46]	87.9	78.3	81.0	103.0	50.8	62.8	91.1	82.4	76.7
+SFD-ADNet (our)	99.4	69.5	86.9	86.9	55.9	68.0	126.0	74.0	39.3

**Table 3:** Training cost analysis.

Method	Augmentation	GPU	Time/Epoch (min) ↓	Peak GPU Memory (GB) ↓	OA (%)
PointNext	Adaptpoint	RTX3070 (12 GB)	3.8	9.6	88.5
PointNext	SDF-ADNet	RTX3070 (12 GB)	7.2	10.8	99.4

**Table 4:** Evaluation of standard part-segmentation model with respect to metric. IoU and object IoU on ShapeNet part benchmark dataset.

Method	Airplane	Bag	Cap	Car	Chair	Earphone	Guitar	Knife	Lamp	Labtop	Motorbike	Mug	Pistol	Rocket	Skate Board	Table	Miou
Point-GR [67]	84.7	83.7	84.0	79.8	-	79.4	91.5	86.5	83.5	95.6	72.7	95.2	82.6	63.02	-	82.3	85.2
GTNet [68]	84.1	80.3	81.5	78.2	90.9	70.2	91.6	87.5	84.8	95.8	61.2	93.9	83.3	53.6	75.4	83.1	85.5
pointNet++	81.9	83.4	86.4	78.6	90.5	64.7	91.4	83.1	83.4	95.1	69.6	94.7	82.8	56.9	76.0	82.3	84.8
+PointWoLF	82.0	83.9	87.3	77.6	90.6	78.4	91.1	87.6	84.7	95.2	62.0	94.5	81.3	62.5	75.7	83.2	85.2
+SFD-ADNet	88.0	79.5	84.8	86.0	86.2	78.0	91.3	90.2	82.5	90.7	78.3	91.7	85.3	74.8	84.7	83.9	84.7
DGCNN	84.0	83.4	86.7	77.8	90.6	74.7	91.2	87.5	82.8	95.7	66.3	94.9	81.1	63.5	74.5	82.6	85.2
+PointWoLF	82.9	73.3	83.5	76.7	90.8	76.7	91.4	89.2	85.2	95.8	53.7	94.0	80.1	54.9	74.3	83.4	85.2
+SFD-ADNet	89.2	80.2	85.5	86.9	87.0	78.9	92.1	91.0	83.2	91.5	79.0	92.5	86.1	75.6	85.5	84.7	85.4

**Table 5:** Experimental results of point cloud attack defense (OA,%).

Method	Perturb ↑	Add-CD ↑	ADD-HD ↑	KNN ↑	Drop-100 ↑	Drop-200 ↑
NoDefense	-	7.24	6.59	-	80.19	68.96
SRS [69]	73.14	65.32	43.11	49.96	64.51	39.60
SOR [70]	77.67	72.90	72.41	61.35	74.16	69.17
SOR-AE [70]	78.73	73.38	71.19	78.73	76.66	68.23
Adv Training [46]	20.03	12.27	10.06	8.63	80.39	67.14
DUP-Net [46]	80.63	75.81	72.45	74.88	76.38	72.00
IF-Defense [71]	86.99	80.19	76.09	85.62	84.56	79.09
AdaptPoint [46]	86.75	80.83	77.03	77.67	86.55	83.59
SFD-ADNet (our)	88.49	81.76	77.52	87.16	85.92	80.61

**Table 6:** Ablation of Mamba encoder (mCE,%).

Method	mCE (↓)
Bi-SSM + LGA	69.5
w/o LGA	72.5
w/o bi-SSM	73.2
Tri-SSM	70.6
One-SSM	71.3

**Table 7:** Ablation of deformation parameter prediction network (mCE, %).

Deformation	Mask	DCDPP	mCE (↓)
×	×	×	92.1
√	×	×	76.4
×	√	×	77.1
×	×	√	78.2
√	√	√	69.5

Note: “√” indicates that the corresponding module is enabled, while “×” indicates that it is disabled.

**Table 8:** Sensitivity analysis of anchor number and loss weight (mCE, %).

Anchor	mCE (↓)	λ	mCE (↓)
2	71.4	0.5	70.4
4	69.5	1	69.5
8	72.6	2	71.1
16	74.3	4	73.2

## Data Availability

The data presented in this study are openly available in the ModelNet40-C dataset at https://github.com/jiachens/ModelNet40-C (accessed on 25 October 2025) and the ScanObjectNN-C dataset at https://github.com/Roywangj/AdaptPoint (accessed on 25 October 2025). The implementation of the method proposed in this work is available at https://github.com/movablebag/SFD-ADNet (accessed on 29 November 2025).
